# Assessing prefrontal cortex activity in Graves’ disease: a functional near-infrared spectroscopy study

**DOI:** 10.3389/fnhum.2025.1559914

**Published:** 2025-04-24

**Authors:** Simon Skau, Mats Holmberg, Birgitta Johansson, Lina Bunketorp Käll, Helge Malmgren, Hans-Georg Kuhn, Helena Filipsson Nyström

**Affiliations:** ^1^Department of Mathematics and Computer Science, Karlstad University, Karlstad, Sweden; ^2^Institute of Neuroscience and Physiology, Sahlgrenska Academy, University of Gothenburg, Gothenburg, Sweden; ^3^Department of Medicine, Huddinge, Karolinska Institutet, Stockholm, Sweden; ^4^ANOVA, Karolinska University Hospital, Stockholm, Sweden; ^5^Wallenberg Center for Molecular and Translational Medicine, Göteborg, Sweden; ^6^Center for Advanced Reconstruction of Extremities (C.A.R.E.), Sahlgrenska University Hospital, Gothenburg, Sweden; ^7^Institute of Medicine, Sahlgrenska Academy, University of Gothenburg, Gothenburg, Sweden; ^8^Department of Endocrinology, Sahlgrenska University Hospital, Gothenburg, Sweden

**Keywords:** Graves’ disease, mental fatigue, fatigability, fNIRS, near-infrared spectroscopy, Stroop, frontal cortex, thyroid

## Abstract

**Introduction:**

Graves’ disease (GD) is associated with cognitive, emotional, and fatigue difficulties. Objective measures of cognitive dysfunction have yielded mixed results. The aim of this study was to investigated whether premenopausal female patients with first-time hyperthyroid GD (mean age 34 years) exhibit cognitive fatigability and altered functional activity in the prefrontal cortex (PFC) using functional near-infrared spectroscopy (fNIRS) during an exhausting cognitive task.

**Methods:**

Using the Animal Stroop test, we compared patients with GD (*N* = 28) and healthy controls (*N* = 28) before and after a 30-min cognitively exhausting reading comprehension task.

**Results:**

Both groups showed improvements in Stroop task performance after the reading task (*p* < 0.001, *η*_p_^2^ = 0.389), and no group differences were observed in cognitive performance. Increased activation in the left dorsolateral prefrontal cortex post-test was found for controls but not for patients with GD. Exploratory analyses showed higher increases in oxy-hemoglobin levels post-test in the PFC of controls compared to patients with GD, indicating reduced PFC involvement in patients with GD.

**Discussion:**

In conclusion, we were not able to show any change in the functional activity of the PFC after prolonged mental activity in this set-up using fNIRS of hyperthyroid GD patients. Further studies are needed to understand the mechanism behind self-reported fatigue in GD.

## 1 Introduction

Graves’ disease (diffuse autoimmune hyperthyroidism; GD) affects ~2,100 people every year in the general population of Sweden (Abraham-Nordling et al., 2011). Patients with GD frequently self-report cognitive problems but it has been challenging to confirm these problems using neuropsychological tests (Vogel et al., 2007; Möller et al., 2017; Ritchie and Yeap, 2015; Johansson et al., 2023). Patients with GD also report mood problems such as depression and anxiety (Ritchie and Yeap, 2015) and pathological fatigue (Johansson et al., 2023; De Leo et al., 2016; Fischer et al., 2018; Holmberg et al., 2022). Here, we denote pathological fatigue as the fatigue that interferes with usual and reasonable desired activities such as work and social life (Skau et al., 2021). Since pathological fatigue has been reported in GD (Johansson et al., 2023; Holmberg et al., 2022), one possibility is that the cognitive dysfunction in GD is related to cognitive fatigability, as being cognitively active over a longer time-period reduces the person’s ability to take part in ordinary activities (Johansson et al., 2009). Cognitive fatigability, defined as the decrement in cognitive performance over a consecutive time interval (Skau et al., 2021), has been difficult to capture in healthy populations (Hagger et al., 2016; Vohs et al., 2021; Inzlicht and Friese, 2019) as well as in individuals suffering from pathological fatigue (Skau et al., 2019; Skau et al., 2021; Möller et al., 2017; Möller et al., 2014; Johansson and Rönnbäck, 2015; Jonasson et al., 2018). Due to the difficulty in capturing fatigue with cognitive tests, researchers have used functional imaging techniques which have shown altered brain activity (Skau et al., 2019; Skau et al., 2021; Berginström et al., 2018; Kohl et al., 2009; Wylie et al., 2017). In two previous studies from our laboratory, one in patients suffering from pathological fatigue after mild traumatic brain injury (TBI) and one in individuals suffering from exhaustion disorder/burn-out syndrome, we could not find any evidence of increasing or decreasing functional activity over 2.5 h in the prefrontal cortex (PFC) (Skau et al., 2019; Skau et al., 2021), a brain area involved during cognitive tasks and part of the frontoparietal (cognitive control) network (Vanderhasselt et al., 2009; Roberts and Hall, 2008). However, in these two studies and compared to controls, both patient groups utilized the PFC differently from the beginning (Skau et al., 2019; Skau et al., 2021). Additionally, when analyzing brain connectivity, we found that functional networks became more segregated after 2.5 h of cognitive activity for patients suffering from pathological fatigue after TBI compared to controls (Skau et al., 2022).

Functional magnetic resonance imaging (fMRI), proton magnetic resonance spectroscopy, and positron emission tomography studies have demonstrated lower activity in the limbic system and frontal, parietooccipital, and temporal lobes in hyperthyroid patients compared to healthy controls (Miao et al., 2011; Schreckenberger et al., 2006; Bhatara et al., 1998; Danielsen et al., 2008; Li et al., 2017; Liu et al., 2020; Zhi et al., 2018; Liu et al., 2017). We recently reported smaller hippocampi in patients with first onset hyperthyroid GD compared to controls, with the reduced volume being normalized after 15 months of treatment (Holmberg et al., 2022). We found no structural effect related to mental fatigue. So far, no study has studied brain activity in patients with GD specifically suffering from fatigue. A commonly applied test of conflict processing in the context of functional imaging is the Stroop task (Roberts and Hall, 2008). The aim was to investigate functional activity using functional near-infrared spectroscopy (fNIRS), a non-invasive optical imaging technique, during conflict processing in the dorsolateral PFC (DLPFC) during two sets of the Animal Stroop task (Szűcs et al., 2012), with a 30 min reading comprehension task in between to study endurable cognitive activity to find biomarkers of the patients’ self-reported fatigue levels in hyperthyroid GD patients. Our hypotheses were that while in hyperthyroidism: (i) patients with GD will have a slower response time for the Animal Stroop task compared to controls; (ii) the difference between the second and first Stroop test will be comparably larger for patients with GD than controls; (iii) patients with GD will have a different functional activity in the DLPFC compared to controls; and (iv) the functional activity of patients with GD will be affected by the reading comprehension task in a different way compared to controls. In addition, we also conducted exploratory correlational analyses between the functional activity of patients with GD and their reported mental health status and hormone levels.

## 2 Methods

### 2.1 Study design

The current study was a sub-study in the CogThy study, a prospective, case-controlled trial at the Endocrine Research Department at Sahlgrenska University Hospital, Göteborg, Sweden, including 65 premenopausal female patients with first-time hyperthyroid GD and 65 matched controls randomly selected from the Swedish population registry after matching for age and sex (Holmberg et al., 2019). The full study protocol has been reported elsewhere (Holmberg et al., 2019). Inclusion criteria were premenopausal, hyperthyroid women with free thyroxine (fT4) levels ≥50 pmol/L (reference range 12–22 pmol/L) or total triiodothyronine (T3) levels ≥6.0 nmol/L (reference range 1.3–3.1 nmol/L). The patients also had to have elevated thyrotropin receptor antibody levels or technetium scintigraphy with a diffuse uptake. Exclusion criteria were pregnancy, serious somatic diseases such as other endocrine diseases, heart failure, respiratory failure, active malignancy, psychosis, inability to follow the study protocol, systemic glucocorticoid treatment (past, present, or anticipated use within 15 months), MRI contraindications such as implants, and amiodarone-induced GD. In the CogThy study, patients were included within 2 weeks of starting treatment with antithyroid drugs (ATDs) while still hyperthyroid. Patients and controls underwent a comprehensive multimodal assessment battery at inclusion, including demographics, smoking status, thyroid hormonal and antibody assessment, psychiatric assessment, and questionnaires for mental fatigue, anxiety, depression, QoL, and neuropsychological assessment. On the same occasion, the participants underwent structural MRI. fNIRS measurements were conducted on a separate day with patients still being in hyperthyroidism. Data on glucoprotein-coupled receptor antibodies in this GD cohort have already been published (Lundgren et al., 2018) as well as data on brain morphology, neuropsychological assessment, and mental sequelae in hyperthyroidism and euthyroidism (Johansson et al., 2023; Holmberg et al., 2022). The current sub-study started in May 2015 and included patient #38 to #65 and their matched controls. The Gothenburg Regional Board of the Swedish Ethical Review Authority approved the study (reference number: T955-14/190-10).

### 2.2 Participants

In this sub-study, 28 patients with GD and 28 controls matched for sex, age, educational level, and smoking habits were included: mean age (SD) was 34.6 (±10.1) and 34.9 (±10.1) years, respectively. The group size was based on a power calculation using data from a study with similar design that involved patients suffering from pathological fatigue after mild TBI (Skau et al., 2019). Since behavioral results has been similar between different patient groups that suffer from pathological fatigue, using data from patients suffering from pathological fatigue after mild TBI was deemed appropriate. With a significance level of 0.05 and a power of 0.8, 23 subjects would be needed in each group to detect a similar difference between groups in oxy-Hb activity in the DLPFC. We decided to include 28 patients and 28 controls in order to allow for loss of data.

### 2.3 Experimental design

**Figure 1:**
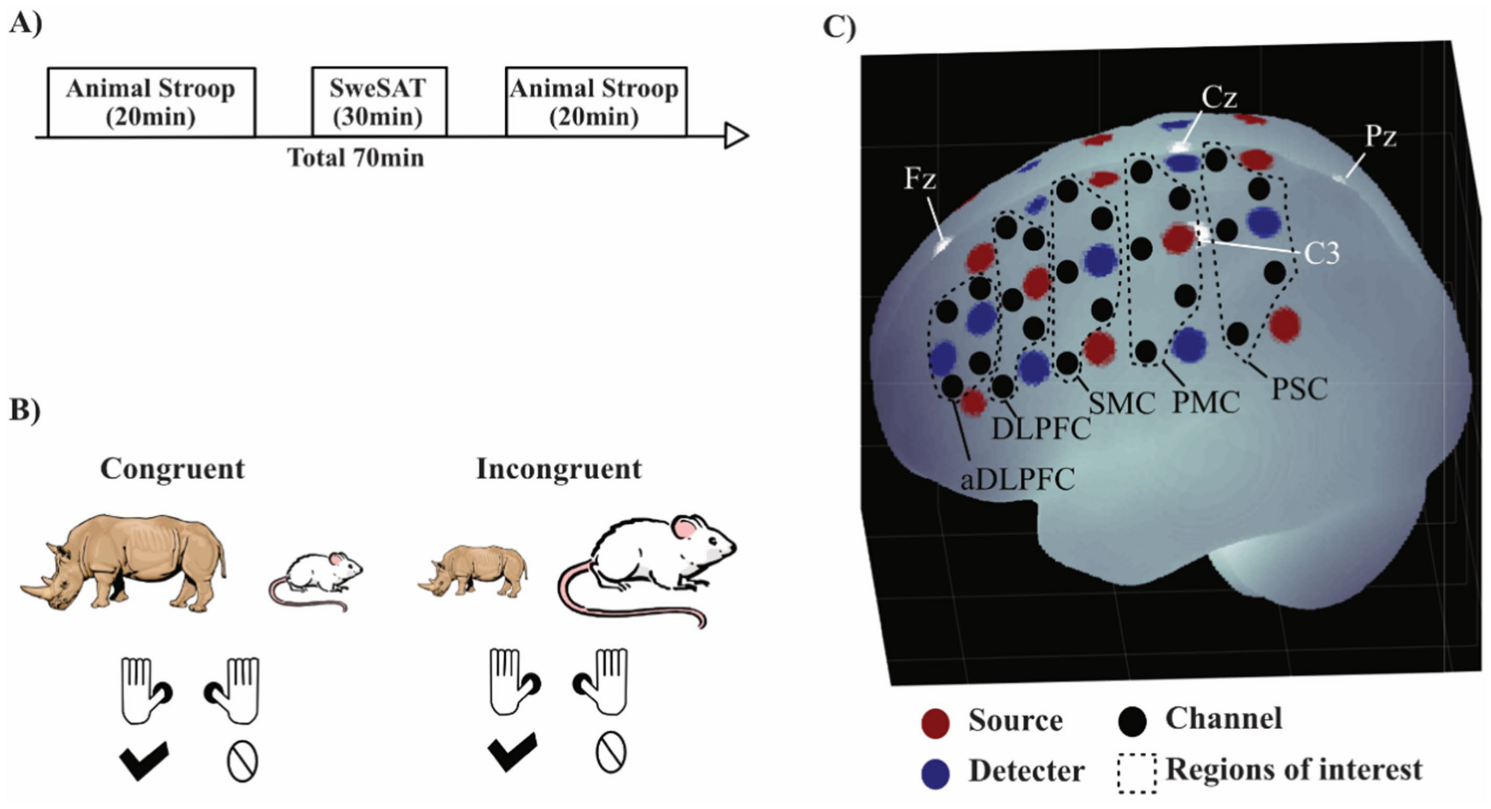
Experimental setup. **(A)** Order of the tests, where SweSAT stands for the reading comprehension task from the Swedish Scholastic Aptitude Test. **(B)** Example of congruent and incongruent trials in the Animal Stroop task. **(C)** Visualization of fNIRS measurements on the left hemisphere. White dots are 10/20 landmarks. Black dashed lines areas indicate regions of interest (ROIs): PSC, primary somatosensory cortex Brodmann area (BA) 3; PMC, primary motor cortex BA 4; SMC, secondary motor cortex BA 6 and 8; DLPFC, dorsolateral prefrontal cortex BA 8 and 9; aDLPFC, anterior DLPFC BA 9. The brain image was generated using a MATLAB-based toolbox (Singh et al., 2005).

fNIRS employs near-infrared light to measure the relative change in oxygenated and deoxygenated hemoglobin (oxy-Hb/deoxy-Hb) a few centimeters into the cortex, as an indirect measure of neural activity (Scholkmann et al., 2014; Tak and Ye, 2014), fNIRS measurements were usually conducted the next day after enrollment but no later than 1 week after the first visit. The participant was seated in a chair next to a table with a computer screen. The fNIRS cap with optodes attached was carefully placed on the participant’s head and worn throughout the experimental sessions. To minimize ambient light, lighting was turned off during the Animal Stroop test. The experiment consisted of three parts: a pre-test, an intermediate task, and a post-test (Figure 1A). For the pre- and post-test, the Animal Stroop test (20 min) was used (Figure 1B). The intermediate part was a reading comprehension test (30 min) taken from the 2010 Swedish Scholastic Aptitude Test (SweSAT), a standardized test used in competitions for a place in courses and programs at Swedish universities. It is made up of five short stories and 20 questions. The task was chosen to induce fatigability in patients, since previous reports indicate that individuals suffering from pathological fatigue have a slower reading speed and experience reading as troublesome and exhausting (Johansson et al., 2009). In the original SweSAT, this part had a time limit of 50 min. Answers on the reading comprehension task were not saved. No fNIRS recording was performed during the SweSAT.

### 2.4 Measurements

#### 2.4.1 Animal Stroop

The Animal Stroop test is a size congruency task (Szűcs et al., 2012), in which the participants are presented with pictures of two different animals simultaneously, one on the left side and one on the right side of the screen (Figure 1B). One of the images is larger on the screen than the other. When the larger in real-life animal is presented as the larger image, it is a congruent trial, while a trial where the larger animal is presented as the smaller image is incongruent. The participants were instructed to answer which of the animals depicted was larger in real life by pressing with their left or right thumb on a gamepad corresponding to the place of the larger in real-life animal on the screen. The test was made up of 80 trials, semi-randomized to generate 20 congruent left-hand response trials, 20 congruent right-hand response trials, 20 incongruent left-hand response trials, and 20 incongruent right-hand response trials to acquire an adequate number of trials for sufficient resolution of the hemodynamic response. The participants were asked to answer as fast and as correctly as possible and had 3 s to respond before the images disappeared from the screen. The time between response and the next stimulus was set to 10–14 s to capture the complete hemodynamic response for each trial (see fNIRS data analysis as follows). The task was divided into four blocks with a 30-s break after each block. Raw scores recorded were response time, errors, and omissions. Each session started with a training session of 20 trials with a short inter-stimulus interval followed by 10 trials with a 12-s inter-stimulus interval.

#### 2.4.2 Mental assessments

Self-evaluation of mental fatigue for the preceding month was performed using the Mental Fatigue Scale (MFS) questionnaire (Johansson et al., 2010). MFS is a validated questionnaire for capturing pathological fatigue in patients following TBI or stroke (Johansson and Rönnbäck, 2013; Johansson et al., 2010). It consists of 15 questions covering symptoms associated with pathological fatigue (Lindqvist and Malmgren, 1990; Lindqvist and Malmgren, 1993) such as rapid mental fatigue upon mental activity, impaired concentration, sensitivity to light and sound, long recovery time, and diurnal variation (Johansson and Rönnbäck, 2013). A total score ≥10.5 indicates deviation from normality (Johansson and Rönnbäck, 2013). Symptoms of depression and anxiety during the week prior to the test session were assessed by self-evaluation with the Comprehensive Psychopathological Rating Scale (CPRS) (Svanborg and Åberg, 1994).

### 2.5 fNIRS acquisitions

fNIRS measurements were performed using a continuous wave system (NTS, Optical Imaging System, Gowerlabs Ltd., United Kingdom) (Everdell et al., 2005) with two wavelengths (780 and 850 nm) to measure changes in the concentration of oxy-Hb, deoxy-Hb, and their sum, total hemoglobin. The system has 16 dual-wavelength sources and 16 detectors. The array generated 48 channels (i.e., source/detector pairs) with a source-detector distance of 30 mm. Optode placement was designed to encompass both the dorsolateral PFC (DLPFC) and motor cortex (Figure 1C) in order to measure a part of the frontoparietal network (Vanderhasselt et al., 2009; Roberts and Hall, 2008). fNIRS data was acquired at a sampling frequency of 10 Hz. The DLPFC was chosen as the region of interests since it is part of the cognitive control network, typically investigated with the Stroop task, and previous studies has found differences in DLPFC for patients suffering from pathological fatigue (Skau et al., 2019).

### 2.6 fNIRS data analysis

fNIRS data were preprocessed using MATLAB 2018b software and the MATLAB-based fNIRS-processing package HomER3 (Huppert et al., 2009). The processing pipeline started with pruning of the data such that channels were rejected if their mean intensity was below the instrument’s noise floor (1 × 10^−4^ AU) followed by converting the raw data to optical densities. A high band-pass filter of 0.05 Hz was used to correct for drift and a low band-pass 0.5 Hz filter to remove pulse and respiration. The HomER3 functions hmrR_MotionArtifactByChannel, with standard deviation threshold set to 30 and amplitude threshold set to 5, and hmrR_MotionCorrectSpline, set to 0.99, were used to correct for motion artifacts. Optical density was converted to hemoglobin concentration by hmrR_OD2Conc, where the partial path length was chosen based on age (Scholkmann and Wolf, 2013). To calculate the hemodynamic response function (HRF), the hmrR_GLM function in HomER3, which estimates the HRF by applying a general linear model (GLM), was used. To solve the GLM, a least-square fit of a convolution model was used (Ye et al., 2009), in which the HRF at each channel and chromophore was modeled with a modified gamma function using recommended values for tau and sigma at 0.1 and 3.0 for oxy-Hb, and 1.8 and 3.0 for deoxy-Hb, respectively.

### 2.7 Statistical analysis

For the confirmatory analysis, i.e., evaluation of our four hypotheses, we used the open-source program JASP version 0.13.1 (Marsman and Wagenmakers, 2017). To evaluate hypotheses (i) and (ii), the response time for correctly answered trials was used as variables in a 3-way repeated ANOVA with one between-group factor *Group* (patients, controls) and two within-group factors, *Stroop effect* (congruent, incongruent) and *Time* (pre, post). The statistic used for the fNIRS data was the beta from the modified gamma function for oxy-Hb from several channels pooled together into predefined regions of interests (ROIs) (Figure 1C). Hypotheses (iii) and (iv) were evaluated with a similar 3-way repeated ANOVA with one between-group factor *Group* (patients, controls) and two within-group factor, *Stroop effect* (congruent, incongruent) and *Time* (pre, post). This was performed separately for oxy-Hb in four ROIs [left/right DLPFC and left/right anterior DLPFC (aDLPFC)]. To correct for multiple comparisons of response time and fNIRS data, a false discovery rate (FDR) was used with the *q*-value set to 0.05 to keep the false positive rate at 5% (Singh and Dan, 2006). A *post-hoc t*-test with Bonferroni correction was also performed.

Two forms of exploratory analysis were performed. First, group differences (GD, control), for all ROIs were analyzed for congruent and incongruent trials during pre- and post-test using independent *t*-tests from MATLAB 2018b. Secondly, correlations (Pearson’s *r* or Spearman *rho* for continuous or ordinal variables, respectively) were calculated between oxy-Hb during incongruent trials and hormone levels [T3, free T3 (fT3), thyroxine (T4), fT4] and mental symptoms (MFS and CPRS).

In the exploratory analysis, no conventional null hypothesis significance testing was performed (where an alpha value is set to control for error rate). Instead, we used a Fisherian interpretation of the statistics *t*, *r*, and *rho* (Perezgonzalez, 2015), where a higher *t*-value, *r*, or *rho* is interpreted as indirect inductive evidence against the null hypothesis (here defined as no difference); the larger the *t*-value, the stronger the evidence. In contrast to the confirmatory analysis, no claims regarding type 1 error rate were made and no correction for multiple comparisons was performed. Under a Fisherian interpretation, it is also possible to use a threshold for “significance.” However, since this terminology is intimately connected to type 1 error control under a null hypothesis significance testing framework and might lead to confusion, we did not apply the term “significance” to our exploratory analysis.

## 3 Results

### 3.1 Descriptive characteristics

Demographic and clinical characteristics of the study population are presented in Table 1.

**Table 1:** Demographic and descriptive results.

	Mean (SD)	
	Patients with GD (*n* = 28)	Controls (*n* = 28)
Age, years	34.6 (10.1)	34.9 (10.1)
Hormone levels		
fT3 (ref: 3.1–6.8), pmol/L	18.7 (12.2)	5.3 (2.0)
fT4 (ref: 12–22), pmol/L	50.3 (26.1)	15.4 (3.3)
T4 (ref: 63–151), nmol/L	187.1 (57.1)	108.3 (25.2)
TSH (ref: 0.3–4.2), mIU/L	<0.014	2.28 (0.98)
Questionnaires		
MFS (<10.5 no pathological fatigue), score	16.6 (6.1)	6.0 (6.4)
CPRS depression, score	7.4 (4.4)	1.9 (2.0)
CPRS anxiety, score	5.7 (4.6)	3.8 (2.5)

GD, Graves’ disease; CPRS, Comprehensive Psychopathological Rating Scale; fT3, free triiodothyronine; fT4, free thyroxine; MFS, Mental Fatigue Scale; ref, reference range; SD, standard deviation; T3, total triiodothyronine; T4, total thyroxine; TSH, thyroid-stimulating hormone.

### 3.2 Behavioral results

**Figure 2:**
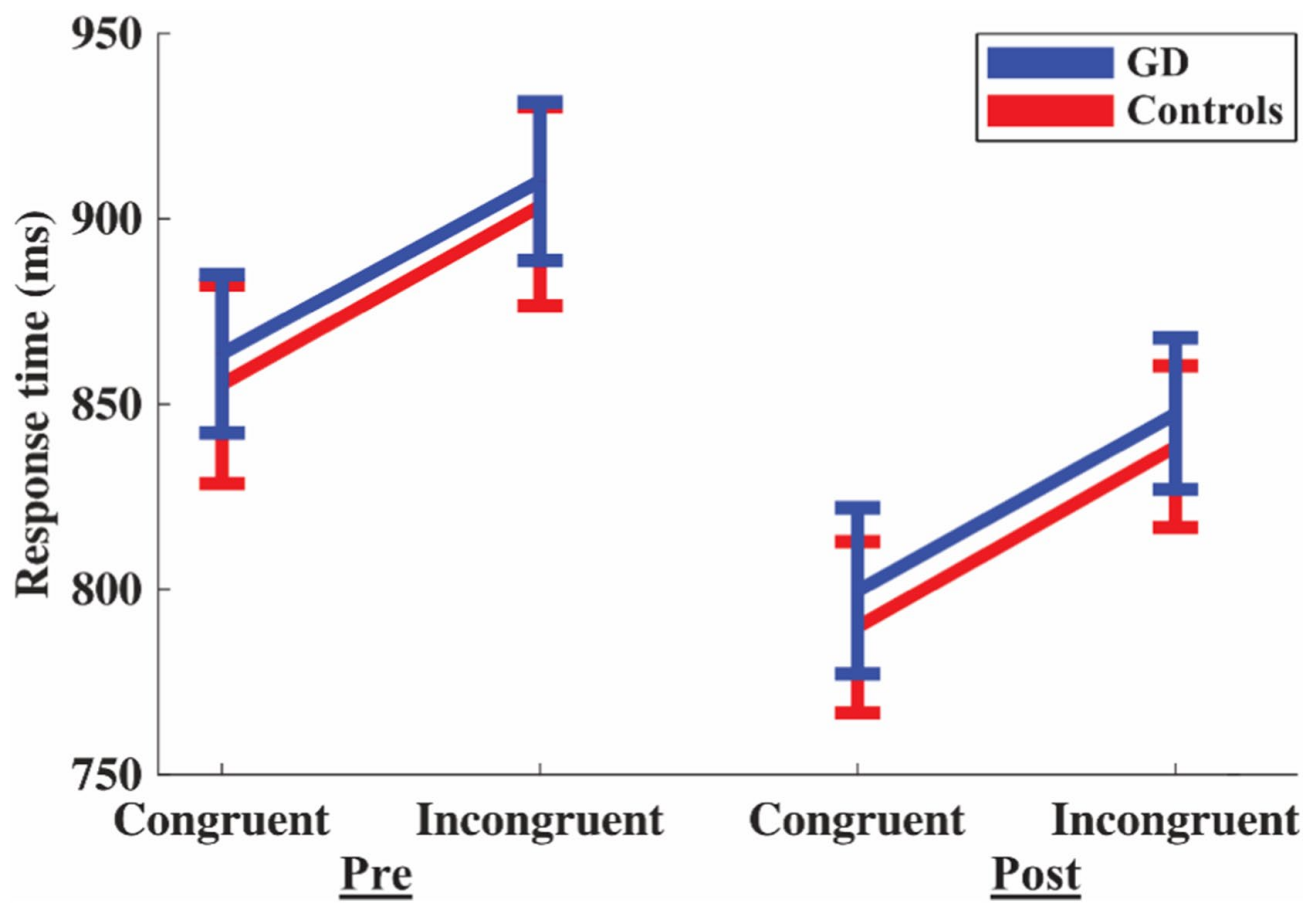
Response time for congruent and incongruent trials during pre- and post-Animal Stroop test. Blue lines are for patients with Graves’ disease (GD) and red lines for controls. Error bars are standard error of the mean.

There was a *Stroop effect*, *F*(1,46) = 69.284 with an FDR-adjusted *p* < 0.001 and an effect size of *η*_p_^2^ = 0.601, indicating that the task manipulations were successful for both groups (i.e., slower on incongruent than congruent trials). There was a *Time effect*, *F*(1,46) = 29.260 with an FDR-adjusted *p* < 0.001 and *η*_p_^2^ = 0.389, indicating that overall response time was faster during the post-session for both groups. Consequently, there was no support for hypothesis (i) (patients with GD will have a worse/slower response time on the Animal Stroop task compared to controls) nor for hypothesis (ii) (the difference between the second and first Stroop test is comparably worse for the patients with GD than for controls). There was no significant difference between the groups, *F*(1,46) = 0.221 with an FDR-adjusted *p* = 1.00 and *η*_p_^2^ = 0.001, no *Stroop* vs. *Group interaction F*(1,46) = 0.089 with an FDR-adjusted *p* = 1.00 and *η*_p_^2^ = 0.002, and no *Time* vs. *Group interaction F*(1,46) = 0.018 with an FDR-adjusted *p* = 1.00 and *η*_p_^2^ < 0.001 (Figure 2). The analysis of accuracy did not show any significant difference with *t*(50) = 0.135 *p* = 0.893 for congruent trials and *t*(50) = 0.155 *p* = 0.877 for incongruent trials during the pretest, as well as *t*(50) = 1.372 *p* = 0.176 for congruent trials, and *t*(50) = −0.285 *p* = 0.777 for incongruent trials during the posttest.

### 3.3 fNIRS results

Hypothesis (iii) (that, while in hyperthyroidism, patients with GD would have a different functional activity in the DLPFC compared to controls) was not supported by our data from any of the ROIs: no *Group effect* in left DLPFC *F*(1,48) = 0.335 with an FDR-adjusted *p* = 1.00 and *η*_p_^2^ = 0.007, right DLPFC *F*(1,46) = 0.258 with an FDR adjusted *p* = 1.00 and *η*_p_^2^ = 0.006, left aDLPFC *F*(1,50) = 0.446 with an FDR-adjusted *p* = 1.00 and *η*_p_^2^ = 0.012, or right aDLPFC *F*(1,48) = 0.581 with an FDR-adjusted *p* = 1.00 and *η*_p_^2^ = 0.006.

**Figure 3:**
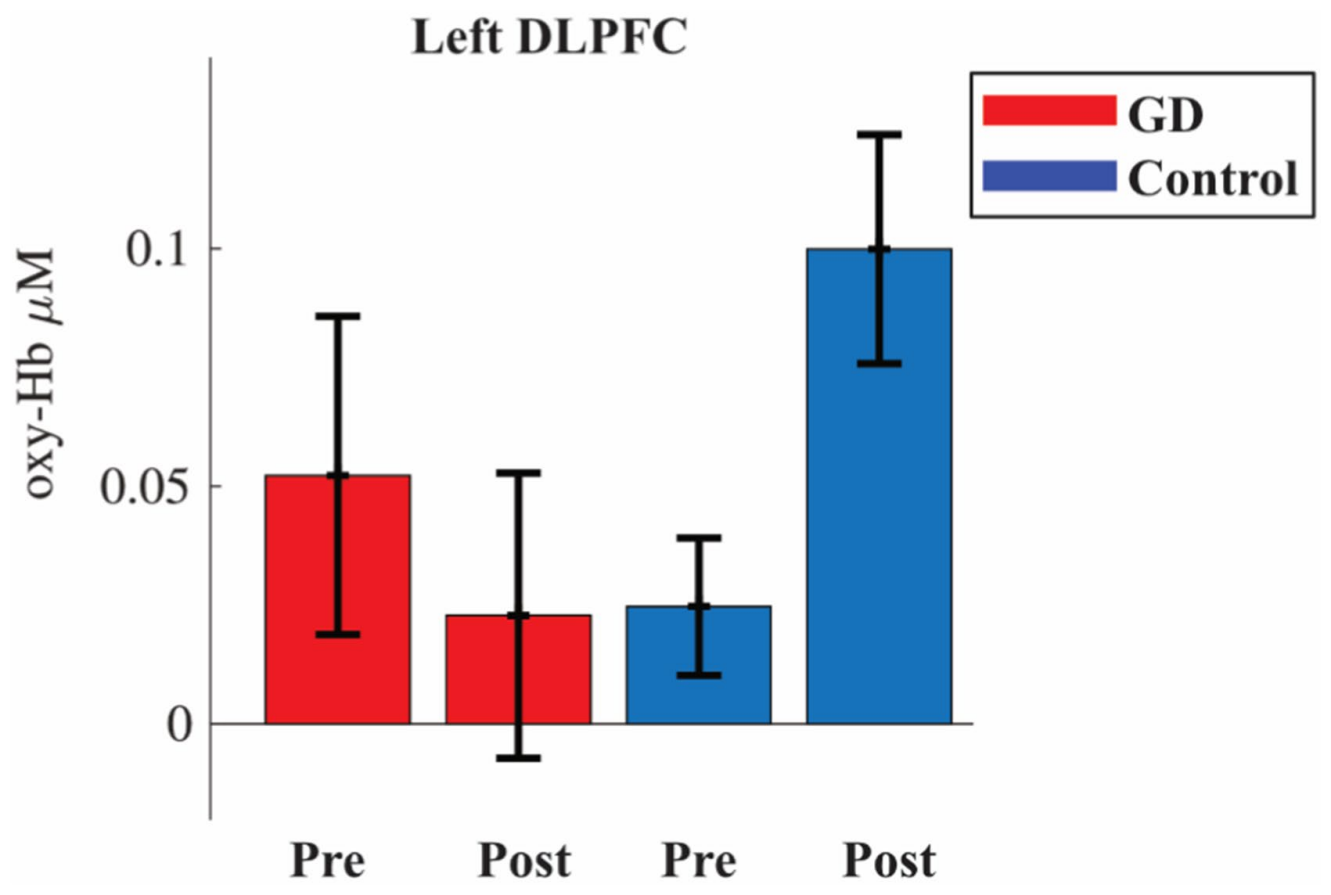
Oxyhemoglobin (oxy-Hb) activity in left dorsolateral prefrontal cortex (DLPFC) during pre- and post-session of the Animal Stroop. Red bars are patients with Graves’ disease (GD) and blue controls. Error bars are standard error of the mean.

Hypothesis (iv) (that while, in hyperthyroidism, the functional activity of patients with GD patient would be affected by the reading comprehension task in a different way compared to controls) was supported for a rejection of the null hypothesis in the left aDLPFC: a *Group* vs. *Time interaction F*(1,50) = 9.785 could be seen with an FDR-adjusted *p* = 0.0156 and *η*_p_^2^ = 0.164. The *post hoc* test showed that the controls had an increased activity in left aDLPFC after the reading comprehension task: *t*(27) = −3.224 with *p* = 0.013 after Bonferroni correction (Figure 3). For the other ROIs, there was no *Group* vs. *Time interaction*: left DLPFC *F*(1,48) = 1.398 with an FDR-adjusted *p* = 1.00 and *η*_p_^2^ = 0.028, right DLPFC *F*(1,46) = 1.621 with an FDR-adjusted *p* = 1.00 and *η*_p_^2^ = 0.034, nor right aDLPFC *F*(1,48) = 1.974 with an FDR-adjusted *p* = 1.00 and *η*_p_^2^ = 0.039 (see Supplementary Table S1 for remaining statistics).

**Figure 4:**
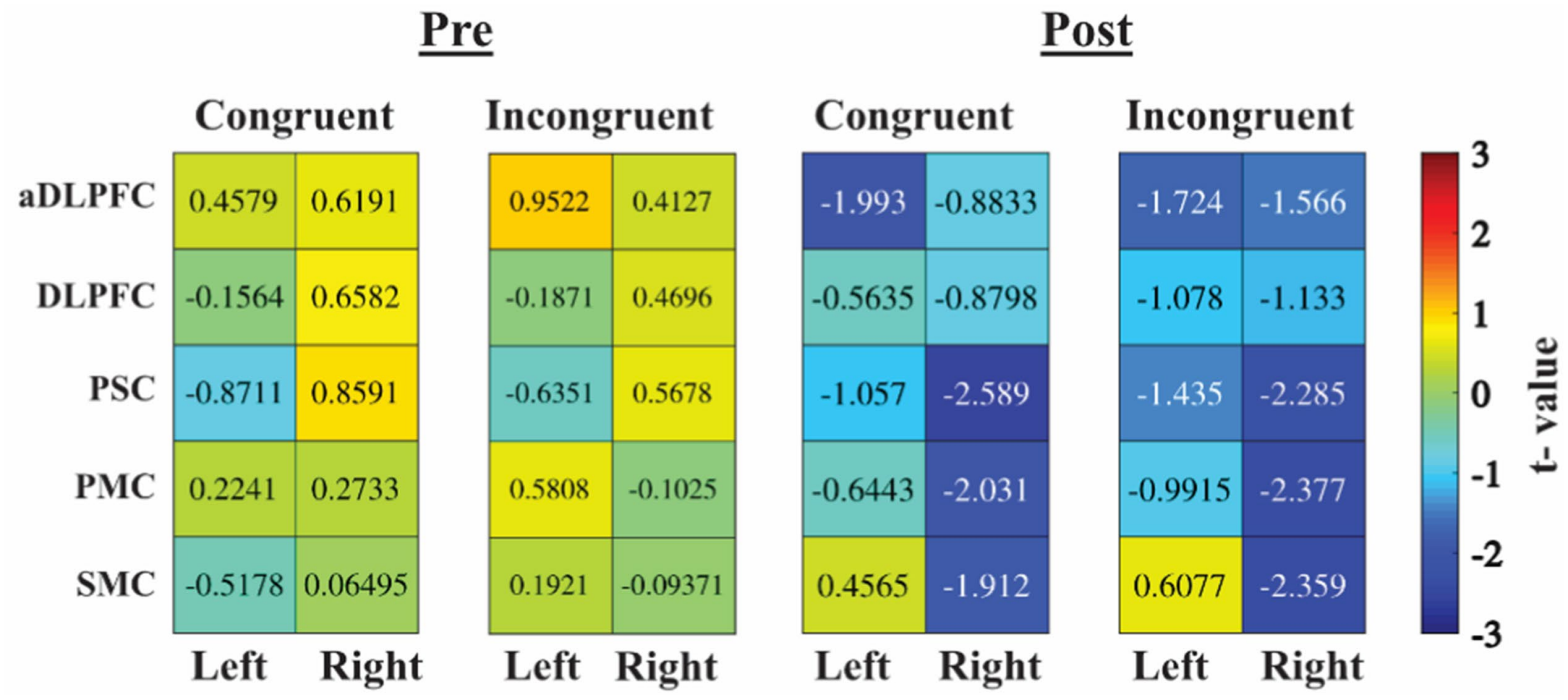
Heatmaps are based on *t*-values from independent *t*-tests. Positive value (warm color/red) is higher oxy-hemoglobin increase for patients with GD compared to controls, negative value (cold color/blue) less oxy-Hb increase for patients with GD compared to controls. aDLPFC, anterior DLPFC; DLPFC, dorsolateral prefrontal cortex; PMC, primary motor cortex; PSC, primary somatosensory cortex; SMC, secondary motor cortex.

We performed two types of exploratory analysis: firstly, group differences for all ROIs and, secondly, correlations between functional activity during incongruent trials and hormone levels, MFS, and CPRS. For the group comparisons, the results from the independent *t*-test are visualized in Figure 4.

**Figure 5:**
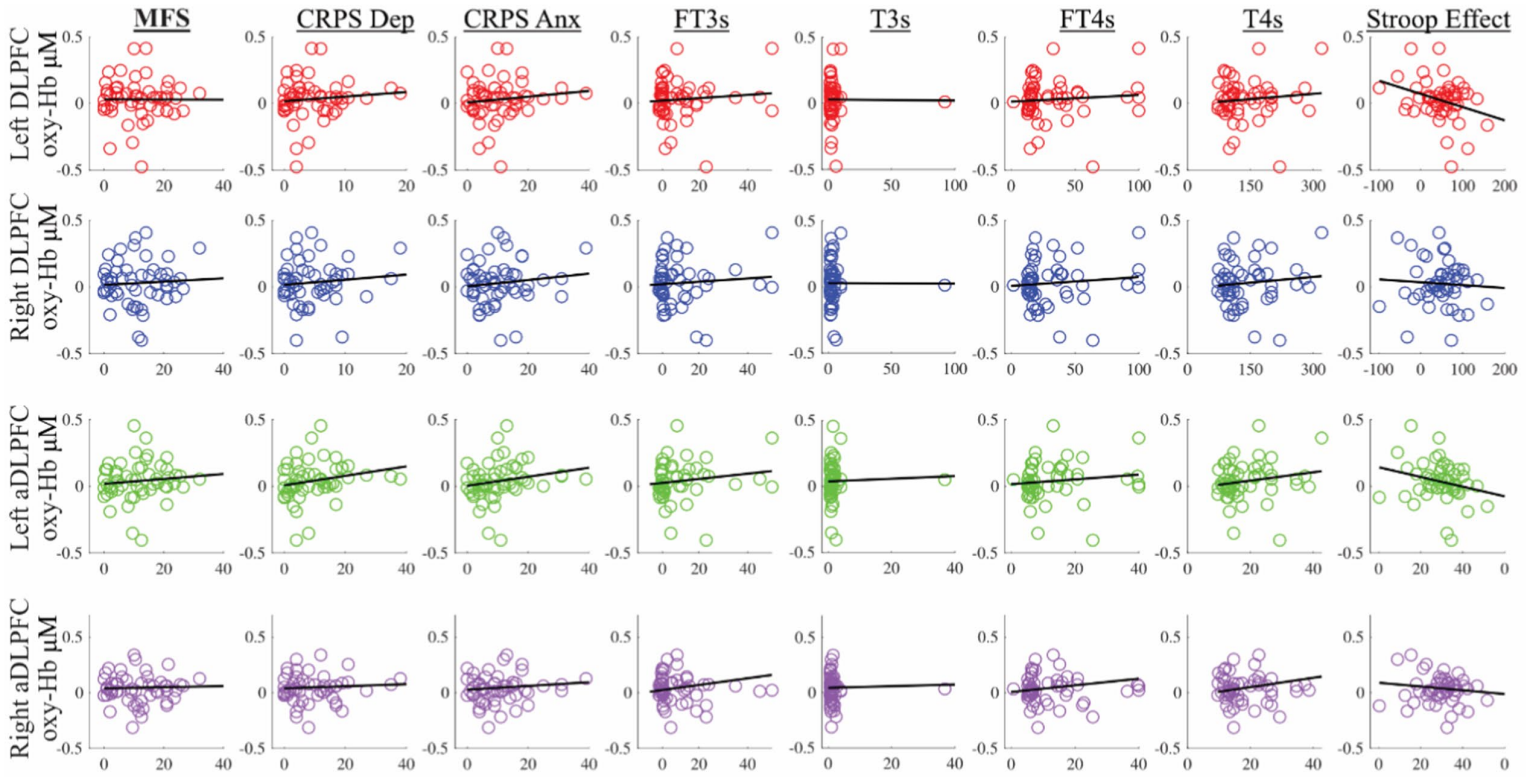
Scatterplots for oxyhemoglobin (oxy-Hb) in micromolar concentration on the y-axis for left/right DLPFC/aDLPFC. Stroop effect response time difference: incongruent-congruent in milliseconds. aDLPFC, anterior DLPFC; Anx, anxiety; CRPS, Comprehensive Psychopathological Rating Scale; Dep, depression; DLPFC, dorsolateral prefrontal cortex; fT3s, free triiodothyronine; fT4s, free thyroxine; MFS, Mental Fatigue Scale; T3s, total T3; T4s, total T4.

The control group showed more activity post-test in the right primary and second motor area and in the DLPFC, but also in the right primary somatosensory cortex (PSC), primary motor cortex (PMC), and secondary motor cortex (SMC), whereas there was no large difference pre-test. In Figure 5, scatter plots for the comparison between oxy-Hb for incongruent trials during the pre-test in left/right DLPFC/aDLPFC are shown. Overall, there were no associations between functional activity and hormone levels, MFS, or CPRS. The analysis did show an association between oxy-Hb during incongruent trials and the Stroop effect (incongruent–congruent trials) (see Supplementary Table S2 for *rho* and *r* values).

## 4 Discussion

In the present study, we investigated whether cognitive performance with the Stroop test and functional activity in the PFC were affected by prolonged mental activity (pre- vs. post-test) in patients with GD compared to controls. Both groups had a longer response time for the incongruent task, indicating that the test *per se* was reliable. In addition, both patients with GD and controls improved their performance in the Animal Stroop task after a 30-min reading comprehension task. This may indicate a Stroop task learning effect in both groups since, contrary to our second hypothesis, the reading comprehension test did not yield any difference in cognitive performance between the groups. Of our four hypotheses, the confirmatory analysis only supported hypothesis (iv) (that while in hyperthyroidism, the functional activity of patients with GD would be affected by the reading comprehension task in a different way compared to controls) with an increased activity for the controls in left DLPFC post-test but not for patients the GD (Figure 3). The exploratory analysis showed evidence for a higher post-test increase in oxy-Hb in the PFC for controls compared to patients with GD (Figure 4).

Previous investigations of the possible cognitive dysfunction associated with GD have given mixed results (Vogel et al., 2007; Möller et al., 2017; Ritchie and Yeap, 2015) where neither Möller et al. (2017) nor Vogel et al. (2007) could show any difference between patients with GD and controls regarding the Stroop test. In the CogThy study, we have previously reported no significant difference between hyperthyroid patients with GD and controls for any of the standard neuropsychological tests used (Johansson et al., 2023). However, in the CogThy study, 89% of the GD participants reported mental fatigue (on MFS) above cut-off compared to 14% of controls. Furthermore, the subjective experience of cognitive symptoms reported with items from MFS (concentration, memory, and slowness of thinking) and the results from cognitive tests differed markedly, indicating that the cognitive tests do not capture the mental fatigue from which the patients suffer (Johansson et al., 2023). In addition, attempts to objectively measure cognitive fatigability, defined here as reduced performance in a cognitive test failed (Hagger et al., 2016; Vohs et al., 2021; Dang et al., 2021). Regarding previous studies in individuals with pathological fatigue, the evidence for cognitive fatigability is also not clear (Skau et al., 2019; Skau et al., 2021; Möller et al., 2017; Möller et al., 2014), which is in accordance with our study. The failure to induce a reduced performance in cognitive tests could be because one or more of our auxiliary hypotheses are false (Meehl, 1990) or because the experimental design of our study was not optimal for showing cognitive fatigability, even in patients suffering from pathological fatigue as identified by the MFS. The hypothetic assumption that pathological fatigue will be induced by a cognitively exhausting task may be false. We know from clinical experience that patients with GD report exhaustion after mental activities that they were previously able to accomplish. Employment often involves many of the most tiresome activities and, among patients with GD, there is a reduced ability to resume work combined with reduced wellbeing compared to that before becoming ill (Fahrenfort et al., 2000).

Hypotheses (iii) and (iv), respectively, that patients with GD have deviant functional activity in the DLPFC compared to controls and that the functional activity in patients with GD is affected by the reading comprehension task in a different way compared to controls, were not supported by our study. In contrast to Göbel et al. (2016), we could not find any group difference in the PFC. We only found support for hypothesis (iv) in the left DLPFC with higher activity for the control group during the post-task. DLFPC is part of the frontoparietal (cognitive control) network involved in solving the Stroop test (Vanderhasselt et al., 2009; Roberts and Hall, 2008). This could indicate that the patients with GD used the frontoparietal network differently than controls in the post-test. However, this was not linked to any difference in our behavioral data. In studies with pathological fatigue after TBI and exhaustion disorder (Skau et al., 2019; Skau et al., 2021), we have reported that patients utilize the DLPFC less during the Stroop test and this already during the pre-test with the effect remaining after the post-test. Studies with the subtraction method, i.e., investigations of more or less activity in specific brain areas, has shown mixed results in patient groups suffering from pathological fatigue (Skau et al., 2019; Skau et al., 2021; Möller et al., 2017). On the other hand, studies of functional networks have found change in connectivity in DLPFC for those suffering from mental fatigue (Skau et al., 2022; Pravatà et al., 2016). Change in functional networks has also been reported in several studies with patients with GD (Li et al., 2017; Liu et al., 2020; Liu et al., 2017), such as networks becoming more segregated, i.e., different brain regions are less functionally connected with each other. This is common among patients after a stroke (Baldassarre et al., 2016) or with multiple sclerosis (Fleischer et al., 2019).

From the exploratory analysis, there was support for a lack of involvement of the PFC in the post-test for the patients with GD but this needs to be studied further in future studies. In addition, we could not find any correlation of functional activity with fT3 or fT4. From our study and reviewing the literature, patient with GD do not seem to have a problem with conflict processing as studied by the Stroop task.

### 4.1 Limitations

By design, the study only included women, which means that any generalization to men should be undertaken with caution. The inclusion of only women may be regarded as a strength as well as a weakness. When evaluating differences in brain function, the restriction to one sex excludes a possible confounder. Also, men and women have different prevalences regarding fatigue-related psychiatric conditions such as depression (Kendler et al., 2006; Rorsman et al., 1990) and the focus on women makes the study more relevant for the group that most affected by GD.

We lacked any estimation of actual experience of fatigue, such as using a visual analog scale directly before and after the experiment. Such a measure would have helped to evaluate the success of the fatigability intervention. The fNIRS measurement did not include any short separation channels used to control blood flow in the scalp and skull, i.e., we were not able to remove irrelevant information from the signal resulting in a hypothetical lower signal to noise ratio, which could have affected the possibility to detect an effect. Due to the limited number of fNIRS optodes, we lacked measurement over the ventrolateral PFC and the posterior parietal cortex, which are essential for cognitive control. In addition, for the power analysis, no studies of patients with GD were found for this analysis and we used data from a fNIRS study that included patients with mild TBI who all suffered from mental fatigue above cut-off in the MFS. The mean value on MFS was higher for mild TBI and lower for controls in that study compared to our GD study. Also, another version of the Stroop test was used (Skau et al., 2019). These differences may have rendered an underestimation of the effects investigated in this study.

Suggestions for future studies is thus; to include not only women, to add additional manipulation checks such as a visual analog scale before and after the experiment, to add short separation channels and to focus more on the whole frontal cortex.

## 5 Conclusion

We could not find any evidence that women with GD were affected by cognitive fatigability induced by prolonged reading and the Stroop test. However, failing to find a statistically significant effect does not mean there is no effect as it might indicate a limitation in sample size, task sensitivity, or methodology. In addition, we were not able to show any change in the functional activity of the PFC after prolonged mental activity. However, these results should be interpreted with caution since we lacked short separation channels and only investigated one cognitive control task. Similar to other patient populations that suffer from pathological fatigue, the patients with GD did not utilize the PFC post-task when solving conflict processing tasks in the same way as healthy controls. Very little is known about how GD affects functional activity of the brain. This study is not about significant clinical effect, it is an exploratory study with the intention to better understand whether the brain is involved and the origin of the cognitive and fatigue problems patient report. Future studies should investigate the DLPFC and other areas of the cognitive control network in patients with GD both with and without pathological fatigue.

## Data Availability

The datasets presented in this article are not readily available because of ethical restriction. Requests to access the datasets should be directed to simon.skau@kau.se.
