# Supplementary material for: Assessing prefrontal cortex activity in Graves’ disease: a functional near-infrared spectroscopy study
Source: Front Hum Neurosci. 2025 Apr 24;19:1559914. doi: 10.3389/fnhum.2025.1559914 (PMC12058655; doi:10.3389/fnhum.2025.1559914)
Supplement: Supplementary file 1 [file Table_1.docx]

***Supplementary Material***

**Assessing Prefrontal Cortex Activity in Graves' Disease: A Functional Near-Infrared Spectroscopy Study**

**Simon Skau*, Mats Holmberg, Birgitta Johansson, Lina Bunketorp Käll, Helge Malmgren, Hans-Georg Kuhn, Helena Filipsson Nyström**

*** Correspondence:**Simon Skau
[simon.skau@kau.se](mailto:simon.skau@kau.se)

SUPPLEMENTARY TABLE S. Result repeated ANOVA for oxy-Hb.

|  | **Cases** | **Sum of squares** | **df** | ***F*** | ***p*** | ***η_p_²*** |
| --- | --- | --- | --- | --- | --- | --- |
| Left DLPFC | Group | 0.034 | 48 | 0.335 | 0.565 | 0.007 |
| Right DLPFC | Group | 0.017 | 46 | 0.258 | 0.614 | 0.006 |
| Left aDLPFC | Group | 0.037 | 50 | 0.589 | 0.446 | 0.012 |
| Right aDLPFC | Group | 0.018 | 48 | 0.310 | 0.581 | 0.006 |
| Left DLPFC | Stroop | 0.001 | 48 | 0.396 | 0.532 | 0.008 |
| Right DLPFC | Stroop | 1.627e–4 | 46 | 0.066 | 0.798 | 0.001 |
| Left aDLPFC | Stroop | 1.343e–4 | 50 | 0.042 | 0.839 | 8.356e –4 |
| Right aDLPFC | Stroop | 4.226e–5 | 48 | 0.025 | 0.874 | 5.289e –4 |
| Left DLPFC | Stroop ✻ Group | 0.003 | 48 | 0.760 | 0.388 | 0.016 |
| Right DLPFC | Stroop ✻ Group | 4.097e–4 | 46 | 0.167 | 0.685 | 0.004 |
| Left aDLPFC | Stroop ✻ Group | 0.003 | 50 | 0.807 | 0.373 | 0.016 |
| Right aDLPFC | Stroop ✻ Group | 0.001 | 48 | 0.753 | 0.390 | 0.015 |
| Left DLPFC | Time | 0.083 | 48 | 2.916 | 0.094 | 0.057 |
| Right DLPFC | Time | 0.031 | 46 | 1.008 | 0.321 | 0.021 |
| Left aDLPFC | Time | 0.032 | 50 | 2.049 | 0.159 | 0.039 |
| Right aDLPFC | Time | 0.029 | 48 | 1.335 | 0.254 | 0.027 |
| Left DLPFC | Time ✻ Group | 0.040 | 48 | 1.398 | 0.243 | 0.028 |
| Right DLPFC | Time ✻ Group | 0.050 | 46 | 1.621 | 0.209 | 0.034 |
| Left aDLPFC | Time ✻ Group | 0.154 | 50 | 9.785 | 0.003 | 0.164 |
| Right aDLPFC | Time ✻ Group | 0.042 | 48 | 1.974 | 0.166 | 0.039 |
| Left DLPFC | Stroop ✻ Time ✻ Group | 0.005 | 48 | 1.235 | 0.272 | 0.025 |
| Right DLPFC | Stroop ✻ Time ✻ Group | 1.157e–4 | 46 | 0.025 | 0.875 | 5.465e –4 |
| Left aDLPFC | Stroop ✻ Time ✻ Group | 4.152e–4 | 50 | 0.121 | 0.729 | 0.002 |
| Right aDLPFC | Stroop ✻ Time ✻ Group | 0.001 | 48 | 0.417 | 0.522 | 0.009 |

aDLPFC, anterior DLPFC; DLPFC, dorsolateral prefrontal cortex; oxy-Hg, oxyhemoglobin.

SUPPLEMENTARY TABLE S2. *Post-hoc* test.

| ***Post-hoc* comparisons**  **Group ✻ Time** | | **Mean difference** | **SE** | ***t*** | ***p_bonf_*** |
| --- | --- | --- | --- | --- | --- |
| GD, Pre | Control, Pre | 0.028 | 0.039 | 0.708 | 1.000 |
|  | GD, Post | 0.029 | 0.025 | 1.200 | 1.000 |
|  | Control, Post | –0.052 | 0.039 | –1.325 | 1.000 |
| Control, Pre | GD, Post | 0.002 | 0.039 | 0.048 | 1.000 |
|  | Control, Post | –0.079 | 0.025 | –3.224 | 0.013 |
| GD, Post | Control, Post | –0.081 | 0.039 | –2.082 | 0.245 |

*Note 1. P*-value adjusted for comparing a family of 6.

*Note 2.* Results are averaged over the levels of Stroop.

*p_bonf_*, Bonferroni-corrected *p*; GD, Graves' disease; SE, standard error.
